# The impact of having a free community eye clinic located inside a homeless shelter: a retrospective analysis of patient demographics

**DOI:** 10.3389/fpubh.2023.1284748

**Published:** 2023-10-13

**Authors:** Amit Ahluwalia, David Morcos, Peter Koulen

**Affiliations:** ^1^The University of Missouri – Kansas City School of Medicine, Kansas City, MO, United States; ^2^Department of Ophthalmology, Vision Research Center, The University of Missouri – Kansas City School of Medicine, Kansas City, MO, United States

**Keywords:** eye care, free eye clinic, homeless shelter, student-run, urban health, vision correction, vision screening

## Abstract

Worsening vision is a life-altering process that affects individuals in many aspects of daily life. While worsening vision can be caused by normal physiological processes that occur with age, there can be underlying systemic or ocular diseases that may be the root cause. Routine eye exams can screen for disease as well determine the degree of vision correction required to attain acceptable vision. Access to an eye exam ordinarily requires vision insurance and one must consider the added expense of glasses if they are recommended. While this can be a life-improving visit for many, there are several socioeconomic barriers that discourage homeless and low-income individuals from being able to access this service. The lack of resources to access regular eye exams and the resulting inadequate eye care may lead to underdiagnosis of serious ocular pathology. The Kansas City Free Eye Clinic is located inside a homeless shelter and, therefore, provides a convenient location for homeless and low-income individuals to receive comprehensive eye exams as well as prescription glasses at no cost. In this paper, we discuss the unique setup and demographics of this student-run eye clinic and the ways in which it has served the Kansas City population and how its integration into a homeless shelter could serve as a role model for free community eye clinics.

## 1. Introduction

As the national rate of homelessness and poverty continues to grow, there has been a shift to emphasize the importance of medical resources for routine health maintenance. In the United States, an estimated 582,000 individuals are currently homeless (1). It is well established that homeless populations have an increased risk for infectious disease, dental problems, mental illness, chronic obstructive pulmonary disease, and cardiovascular disease (2). This therefore necessitates resources that offer free to low-cost health services to the vulnerable homeless population. In terms of eye care, this translates to regular eye exams to screen for refractive errors, ocular-specific pathologies, and ocular manifestations of systemic disease.

The American Academy of Ophthalmology recommends that a routine comprehensive eye exam is completed once every year for healthy individuals with no symptoms of vision problems starting at age 40 (3). For those individuals 60 or older, it is recommended to have a complete eye exam every year or more often if someone wears glasses or contact lenses, has a family history of eye disease or loss of vision, has a chronic disease that puts them at greater risk of eye disease, such as diabetes, or takes medications that have potential ocular side effects (3).

The cost of an eye exam can be an obstacle to routine eye care. A recent CDC survey illustrated that the most common reason (39.8%) for not seeking eye care among those with moderate-to-severe visual impairment was cost or lack of insurance (4). In the Midwest, the average cost of a complete eye exam is currently $39 at an optometrist office and $54 at ophthalmologist office (5). While routine eye exams are important, they are oftentimes not affordable to vulnerable populations. However, it is of utmost importance to receive proper care as poor visual acuity and several ocular conditions have a significant impact on an individual’s well-being as well as earning potential (6). Among the homeless population, there are special considerations that necessitate greater eye care. Regarding ophthalmic patients in an inpatient setting, trauma-related diagnoses in the homeless population was greater compared with the general population at 38 percent and 23 percent, respectively (7). In an additional study, systemic disease was found to be more prevalent in homeless populations that presented to free clinics: self-reported diabetes (17.1%) was found to be significantly higher than that of the general population (7.5%), and fewer than one-third of diabetic participants had ever been evaluated by an ophthalmologist (8). In the same study, a wide variety of undiagnosed retinal pathologies were discovered by slit-lamp examination including diabetic retinopathy, retinal hypertension, epiretinal membrane, drusen, and nevus (8). Missing routine eye exams can lead to underdiagnosis of sight-threatening conditions that remain silent until later, more difficult to treat stages of such diseases (9).

Additionally, the race distribution of the national homeless population is quite different from the overall population. In the United States, African-Americans make up 13% of the overall population but 37% of the homeless population. Similarly, Latino-Americans make up 19% of the overall population but 24% of the homeless population (10). In terms of age, 75% of homeless individuals nationwide are 24 years of age or older (10). Notably, certain risk factors such as age and race can increase the need for an eye exam. According to the American Academy of Ophthalmology, African and Latino-Americans have a much higher risk of developing diabetic retinopathy, glaucoma and cataracts (11). Age is also a predisposing or contributing factor in diabetic retinopathy, glaucoma, cataracts, macular degeneration and should be taken into consideration for frequency of eye exams (12). Among those who cannot afford eye care, volunteer-based free clinics offer, therefore, a much-needed service.

National surveys have illustrated the importance of volunteer-based free clinics: according to patients, if their current free clinic did not exist, 24% would not seek care, 47% would attempt to seek care at another free clinic, and 23% would present to the emergency room (4). Free clinics appear to have the greatest impact on vulnerable populations. In another recent national survey among free clinics, 92.2% of patients were uninsured, 41.9% were homeless, and 39.3% were immigrants (13). While the free clinics target a variety of health conditions, among all national clinics, only 34.4% offered eye exams while only 11.1% offered eyeglasses (13). Due to the aforementioned reasons, there is a clear need for clinics that offer a combination of reliable, free to low-cost, routine eye care as well as glasses for those that cannot afford care.

Kansas City has not been immune to the national rise in homelessness; in fact, according to the Greater Kansas City Coalition to End Homelessness, there are an estimated 1,800 homeless individuals on the streets of Kansas City on a given night (14). The Kansas City Free Eye Clinic (KCFEC) serves as a resource to provide free eye exams as well as glasses at no cost to patients. Patients are able to be seen on a walk-in basis and are checked in by a team of trained volunteers who perform a basic eye evaluation including a peripheral vision test, ocular motility, pupillary reactions, visual acuity, autorefractor analysis, and intraocular pressure. The patient then receives a comprehensive dilated slit lamp exam by a board certified optometrist to screen for pathology as well as evaluate refractive error. If refractive error is present, patients are eligible to receive custom prescription eyeglasses and choose from a variety for frames, free of cost. The patients are fitted for lenses and are able to pick up their new eyeglasses in 1 to 2 weeks. At the time of receiving glasses, patients are able to evaluate their satisfaction with visual acuity and adjustments can be made as needed. The reliable location of the eye clinic provides ease when picking up glasses and assists in repairing or ordering new glasses if previous pairs are damaged. The clinic has also evolved to accommodate age-related vision change by offering free reading glasses, a service that has offered help to many individuals.

Location that allows for continuity of care is key for reaching vulnerable patient populations at free clinics (15). Continuity of care is associated with increased patient satisfaction, increased take-up of health promotion, greater adherence to medical advice and decreased use of hospital services (16). Currently, the majority of free clinics are located in hospitals and churches at 31.6 and 26.3%, respectively, while free clinics in homeless shelters represent a small minority (12). Only 10.5% of free clinics nationwide were reported to be located inside a homeless shelter (12). The KCFEC is unique because it is part of the minority of clinics located inside a homeless shelter along with the fact that it is part of an even smaller minority of free clinics that provides a combination of eye exams, eyeglasses, and a permanent location for consistency of care. The KCFEC is located in the Hope Faith Homeless Day Center, a shelter that provides services such as food, showers, clothing, laundry service, and case management. This convenient location allows individuals to receive a host of resources that address key social determinants of health in a timely and consistent manner. In addition to convenient location, the KCFEC is a venue for education as it allows students to gain valuable experience in patient care. The KCFEC is a student-run clinic that selects motivated students to provide care through community service, patient interviewing, and important visual testing. If students are selected after applying, they receive training by clinic staff and practicing optometrists to ensure that they are proficient in assessing pupil reactivity, ocular pressure, visual acuity, and autorefraction. In this paper, we describe the demographics of the KCFEC, services offered, and return rates of patients. In doing so, we aim to demonstrate the efficacy of the clinic setup and its ability to provide vulnerable populations with free eye care.

## 2. Methods

A retrospective chart review of patient data at the KC Free Eye Clinic from January 2017 to December 2021 was performed. Information including the age, gender, race, language, housing, education, employment, insurance status, government benefits, history of eye trauma, ocular diagnosis, referrals, return rates, impact of poor vision, and alternative to care at KCFEC were collected for each patient at every visit.

The services provided at the clinic were also tracked. Patients were de-identified and data was compiled into the Athenanet Electronic Medical Record. IRB approval was obtained from the University of Missouri- Kansas City IRB (protocol # 2093815) to use data for research purposes. Patient information was compiled into a table showing the age, gender, race, education level, housing type, referral source, employment status, insurance status, disability status, government benefits status, impact of poor vision, eye trauma, and help without KCFEC. The findings were presented in the form of a poster presentation at the (14) annual Society of Public Health Education meeting.

## 3. Results

**Figure 1:**
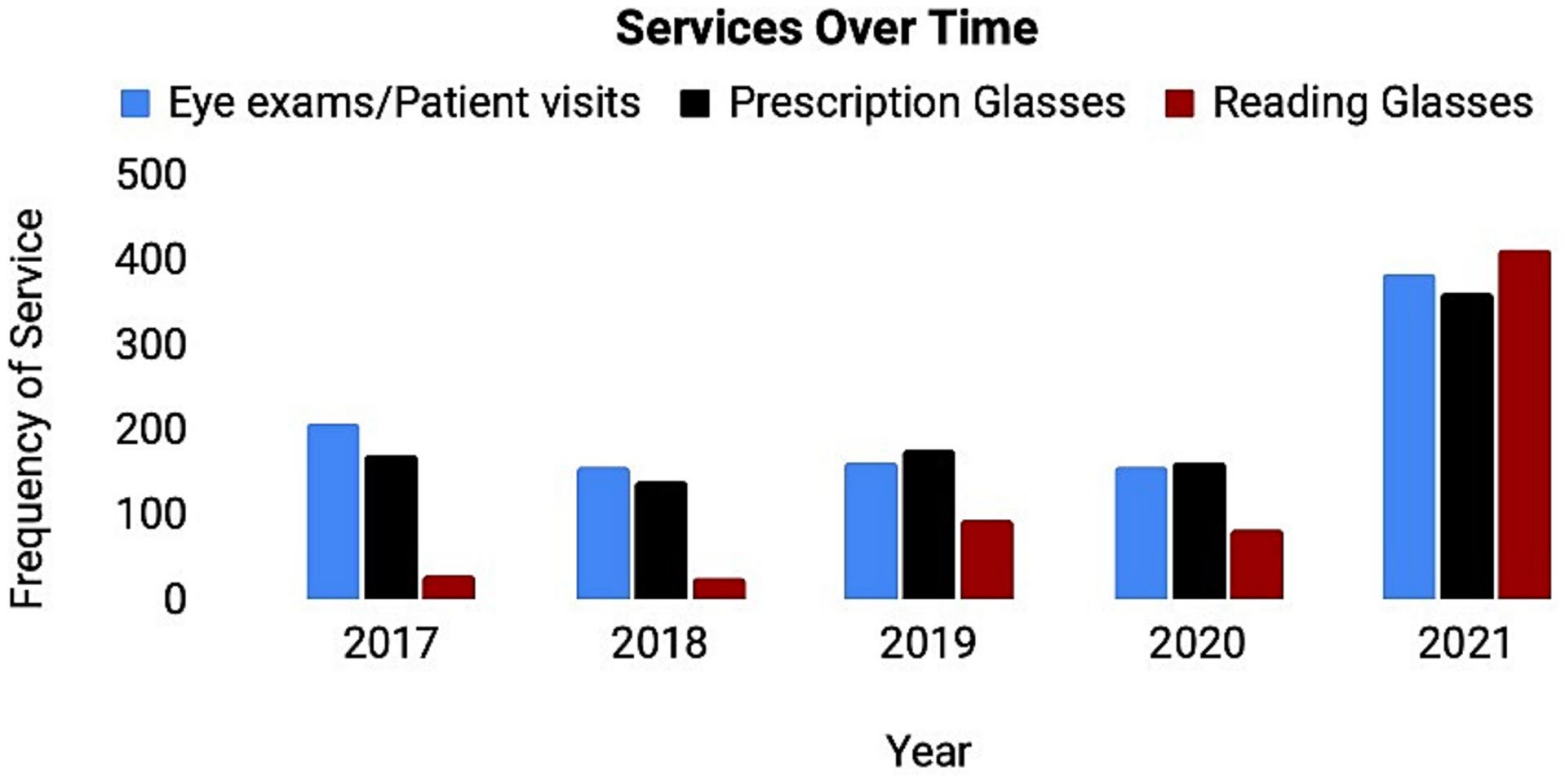
Services offered in the KCFEC from 2017 to 2021. Graphic representation of eye exams (blue), prescription glasses (red), and reading glasses (yellow) provided by KCFEC over time. The number of times the service was performed (*y*-axis) is shown for a given year (*x*-axis) between 2017 and 2021. Data was collected at each visit and summarized for each fiscal year. A notable increase in all services performed can be observed with the largest change being from 2020 to 2021.

The clinic demonstrated tremendous growth from 2017 to 2021. The overall number of eye appointments/ patient encounters, prescription glasses, and reading glasses increased (Figure 1). There was a notable increase from 2020 to 2021 in eye exams/patients encounters, prescription glasses given, and reading glasses given (Figure 1).

384 patient visits occurred in 2021 (Table 1). 2021 was chosen as a point in time analysis as it was the most recent year that data was available to the clinic. The data presented in Table 1 is based on the responses given at patient encounters.

**Table 1:** 2021 Kansas City free eye clinic demographics^a^.

**Total number of patient visits**	**384**
**Gender**	**Percentage of patients** ^**b**^
Male	66.6 (255/384)
Female	33.4 (129/384)
**Age**	**Percentage of patients** ^**b**^
0–20	3.6 (14/383)
21–45	38.1 (146/383)
46–60	43.6 (167/383)
61–75	13.1 (50/383)
75+	1.6 (6/383)
**Race**	**Percentage of patients** ^**b**^
White	40.1 (121/302)
Black	46.7 (141/302)
Hispanic	10.9 (33/302)
Other	2.3 (7/302)
**Housing type**	**Percentage of patients** ^**b**^
Homeless or shelter	67.9 (182/268)
Someone else’s house	13.1 (35/268)
Own house	19 (51/268)
**Referral source**	**Percentage of patients** ^**b**^
Hope Faith (i.e., at the same location)	82.7 (206/249)
Other center	5.7 (14/249)
Independent	11.6 (29/249)
**Education**	**Percentage of patients** ^**b**^
Elementary	0.4 (1/266)
High school incomplete	18 (48/266)
High school complete	43.6 (116/266)
College incomplete	23.7 (63/266)
College complete	12 (32/266)
Masters/Grad school	2.3 (6/266)
**Patient employment**	**Percentage of patients** ^**b**^
Full time	10.9 (29/266)
Part time	7.5 (20/266)
Unemployed	70.7 (188/266)
Unemployed-disability	7.5 (20/266)
Student	0.8 (2/266)
Retired	2.6 (7/266)
**Insurance**	**Percentage of patients** ^**b**^
Uninsured	60 (159/265)
Medicaid	19.6 (52/265)
Medicare	5.7 (15/265)
Medicaid and medicare	7.2 (19/265)
Veterans	2.6 (7/265)
Private insurance	4.9 (13/265)
**Government benefits**	**Percentage of patients** ^**b**^
Yes	55.7 (146/285)
No	44.3 (116/285)
**Impact of poor vision**	**Percentage of patients** ^**c**^
Difficulty to drive/use transportation	17.4 (106/608)
Difficulty reading	31.6 (192/608)
Held back at work/school	5.8 (35/608)
Worried about safety/belongings	11.2 (68/608)
Painful/irritated eyes	11.8 (72/608)
Vision is getting worse/worried about losing vision	22.2 (135/608)
**Eye trauma**	**Percentage of patients** ^**b**^
Yes	20.9 (57/273)
No	79.1 (216/273)
**Diagnoses**	**Number of patients**
Diabetic retinopathy	3
Cataracts	27
Glaucoma/pre-glaucoma	4
Macular degeneration	1
other suspected disease	15
**Help without KCFEC**	**Percentage of patients** ^**b**^
Would not get help	49.6 (133/268)
Would try to pay somewhere	7.5 (20/268)
Would visit ED	3 (8/268)
Would visit other free clinic	39.9 (107/268)
**Return appointments from 2017–2021**	**Number of patients**
2 visits	84
3 visits	14
4 visits	3
5 visits	1

a Demographics were self-reported by patients at each visit in the year 2021. Non-responders were excluded from analysis and percentages were calculated based on available responses.

b Patients/total responses.

c Patients were able to select multiple responses.

Regarding patient demographics, the majority of patients seen at the KCFEC were between the ages of 46 and 60 (43.6%), male (66.6%), and African American (46.7%) (Table 1). The mean age of patients served at the clinic was 46.7 (Table 1).

Regarding socioeconomic status, the majority of patients were unemployed (70.7%), homeless or living in a homeless shelter (67.9%), uninsured (60%), reported using government benefits (65.7%), and completed a high school education (43.6%) (Table 1).

Regarding referrals, the majority of patients (82.7%) were referred to the KCFEC from the Hope Faith Homeless Shelter (Table 1). 49.6% of them stated that they would not receive ophthalmologic care if the KCFEC was not around, and 39.9% reported that they would try to find another free clinic (Table 1).

Regarding medical impact, around 1/5 patients presented with eye trauma (20.9%) (Table 1). Common complaints included difficulty reading (31.6%), anxiety about vision loss (22.2%), and difficulty driving (17.4%) (Table 1). Additionally, a variety of diagnoses were made that impacted future care for patients. Diagnoses included cataracts (27 patients), glaucoma (4 patients), and other various suspected diseases (15 patients) (Table 1).

Regarding continuity of care, the clinic also demonstrated good consistency of care as 102 patients returned to the clinic at least twice from 2017 to 2021. Patients returned up to 5 times between the years of 2017–2021 (Table 1).

## 4. Discussion

In the present study, we attempted to determine the demographics of the KCFEC and its role in serving the Kansas City population with consistent eye care.

A nationwide study (13) on all free clinics in 2010 sent a questionnaire to every national, regional and state free clinic to help highlight various features of free clinics. Free clinics were affiliated with a variety of locations including hospitals and churches at 31.6 and 26.3%, respectively, as well as universities, social service agencies, and medical schools/centers (13). Notably, homeless shelters represented the smallest minority in regards to affiliations at just 10.5%. Of all respondents, only 34.4% of clinics offered eye exams while only 11.1% offered eyeglasses (13).

Our analysis of the KCFEC allowed us to thoroughly investigate the underutilized affiliation of free clinics with homeless shelters. Currently, there is no literature describing the demographics of a free eye clinic affiliated with a homeless shelter to our knowledge. Our data illustrated that the clinic was able to address many vulnerable populations through its various services. The majority of presenting patients were homeless (67.9%) and uninsured (60%). Patients demonstrated a clear need for free eye care (Table 1). A majority of patients responded saying that without the clinic they would not have gotten care otherwise (49.6%) or would have attempted to get care at another free clinic (39.9%) (Table 1). This further highlights the importance of volunteer-based free clinics which are the only viable option for many homeless and uninsured patients to receive any eye care.

Referral source also seemed to be an important factor in how patients presented. The majority of the patients were referred from Hope Faith, the shelter in which the free eye clinic is located (82.7%) (Table 1). The easily accessible location of the clinic seemed to help disadvantaged patients return to the clinic at a tremendous rate. From 2017 to 2021, 102 patients returned to the clinic at least twice, with some patients returning up to 5 times for routine eye appointments (Table 1). There was also a large increase in the number of eye exams, prescription glasses provided, and reading glasses provided from 2020 to 2021 (Figure 1). This increased number potentially resulted from a variety of factors including but not limited to: resurgence of patients after the COVID-19 pandemic, increased volume of individuals at the homeless shelter, and better turnout due to good continuity and rapport with patients.

Providing free eye care is of the utmost importance (4). With such variability in how free clinics approach providing care, exploring differences in location, services offered, and continuity can potentially expose improved ways of reaching target populations. In this study, the demographics at our given clinic shed light on an underutilized location of free clinics: homeless shelters. With strategic location, the clinic was able to evaluate many underrepresented patients for refractive error and provide them with free glasses (Table 1). Additionally, many previously undetected pathologies were diagnosed, including cataracts, diabetes, macular degeneration, and glaucoma (Table 1).

We suggest this unique setup to others attempting to provide free eye care. Those seen at the shelter receive the most up to date information on the hours of operations, are able to establish strong connections with the staff and volunteers at the clinic and have a direct source of communication if there are any questions about their vision health or replacement glasses. As approaches to care evolve, the need for good eye care will continue to persist. When considering location to best serve all, homeless shelters should be taken into consideration for free clinics to establish rapport and provide good, quality care for vulnerable populations.

## 5. Limitations

The present study was a single-center study limited to Kansas City, Missouri during 2017 to 2021. Data was not complete for 2022 and thus not utilized. This limited our data to only 2017 to 2021 with 2021 being the most recent data available, and the year we focused our analysis on. During data collection, some patients elected to omit answers pertaining to the given questionnaire. For this purpose, percentages were used to quantify frequency of responses based on all respondents for a desired measure.

## Data availability statement

The original contributions presented in the study are included in the article/supplementary material, further inquiries can be directed to the corresponding author.

## Ethics statement

The studies involving humans were approved by UMKC IRB (protocol number 2093815). The studies were conducted in accordance with the local legislation and institutional requirements. Written informed consent for participation was not required from the participants or the participants’ legal guardians/next of kin in accordance with the national legislation and institutional requirements.

## Author contributions

AA: Formal analysis, Resources, Supervision, Visualization, Writing – review & editing, Conceptualization, Data curation, Investigation, Methodology, Project administration, Validation, Writing – original draft. DM: Formal analysis, Resources, Supervision, Visualization, Writing – review & editing, Conceptualization, Data curation, Investigation, Methodology, Project administration, Validation, Writing – original draft. PK: Formal analysis, Resources, Supervision, Visualization, Writing – review & editing.

## References

[1] Richard MK Dworkin J Rule KG Farooqui S Glendening Z Carlson S. Quantifying doubled-up homelessness: presenting a new measure using US census microdata. Hous Policy Debate. (2022) 14:1–22. doi: 10.1080/10511482.2021.1981976

[2] Lewer D Aldridge RW Menezes D Sawyer C Zaninotto P Dedicoat M. (2019). Health-related quality of life and prevalence of six chronic diseases in homeless and housed people: a cross-sectional study in London and Birmingham, England. BMJ Open 9:e025192. doi: 10.1136/bmjopen-2018-025192 PMC6501971 31023754

[3] Turbert D. Eye exam and vision testing basics American Academy of Ophthalmology (2023). Available at: https://www.aao.org/eye-health/tips-prevention/eye-exams-101

[4] Gertz AM Frank S Blixen CE. A survey of patients and providers at free clinics across the United States. J Community Health. (2011) 36:83–93. doi: 10.1007/s10900-010-9286-x, PMID: 20532596

[5] Soroka M. Comparison of examination fees and availability of routine vision care by optometrists and ophthalmologists. Public Health Rep. (1991) 106:455–9. PMID: 1908597 PMC1580253

[6] Tielsch JM Sommer A Katz J Quigley H Ezrine S. Socioeconomic status and visual impairment among urban Americans. Baltimore eye survey research group. Arch Ophthalmol. (1991) 109:637–41. doi: 10.1001/archopht.1991.01080050051027, PMID: 2025164

[7] Turkiewicz M Bhandarkar A Arya N Bydon M Shen J. Primary ophthalmic hospitalizations among individuals experiencing homelessness in the United States, 2016–2018. Invest Ophthalmol Vis Sci. (2022) 63:2141–A0169.

[8] Barnes JB Barnes SS Small CR Otto CS Bennett MD. Mobile eye screenings for Hawaii’s homeless: results and applications. Clin Optom. (2010) 12:73–7. doi: 10.2147/OPTO.S13007

[9] Davis TS Luo F Xie SJ Muro-Fuentes EA Rodrigues EB. Evaluating adherence to diabetic retinopathy care in an urban ophthalmology clinic utilizing the compliance with annual diabetic eye exams survey. Cureus. (2023) 15:e34083. doi: 10.7759/cureus.34083, PMID: 36843721 PMC9946894

[10] de Sousa T Andrichik A Cuellar M Marson J Prestera E Rush K. Annual homeless assessment report (AHAR) to congress. The U.S. Department of Housing and Urban Development. (2022). https://www.huduser.gov/portal/sites/default/files/pdf/2022-AHAR-Part-1.pdf (Accessed January 20, 2023).

[11] Apeles L Shin H. Ethnicity and eye disease: a risk reminder for Asian-, African- and Latino-Americans American Academy of Ophthalmology (2016) Available at: https://www.aao.org/eye-health/news/ethnicity-eye-disease-risk-reminder-asian-african-

[12] Centers for Disease Control and Prevention. Healthy aging includes healthy vision Centers for Disease Control and Prevention (2021). Available at: https://www.cdc.gov/visionhealth/resources/features/healthy-aging-vision.

[13] Darnell JS. Free clinics in the United States: a nationwide survey. Arch Intern Med. (2010) 170:946–53. doi: 10.1001/archinternmed.2010.107, PMID: 20548006

[14] Greater Kansas City Coalition to End Homelessness. Annual homeless count: find homeless statistics in Kansas City – greater Kansas City coalition to end homelessness Greater Kansas City Coalition to End Homelessness (2023). Available at: https://gkcceh.org/point-in-time/

[15] Birs A Liu X Nash B Sullivan S Garris S Hardy M. Medical care in a free clinic: a comprehensive evaluation of patient experience, incentives, and barriers to optimal medical care with consideration of a facility fee. Cureus. (2016) 8:e500. doi: 10.7759/cureus.500, PMID: 27014534 PMC4803534

[16] Pereira Gray DJ Sidaway-Lee K White E Thorne A Evans PH. Continuity of care with doctors-a matter of life and death? A systematic review of continuity of care and mortality. BMJ Open. (2018) 8:e021161. doi: 10.1136/bmjopen-2017-021161, PMID: 29959146 PMC6042583

